# Metabolomics of Pregnancy Complications: Emerging Application of Maternal Hair

**DOI:** 10.1155/2018/2815439

**Published:** 2018-12-18

**Authors:** Thibaut D. J. Delplancke, Yue Wu, Ting-Li Han, Lingga R. Joncer, Hongbo Qi, Chao Tong, Philip N. Baker

**Affiliations:** ^1^Department of Obstetrics, The First Affiliated Hospital of Chongqing Medical University, Chongqing 400016, China; ^2^State Key Laboratory of Maternal and Fetal Medicine of Chongqing Municipality, Chongqing Medical University, Chongqing 400016, China; ^3^International Collaborative Laboratory of Reproduction and Development of Chinese Ministry of Education, Chongqing Medical University, Chongqing 400016, China; ^4^Liggins Institute, University of Auckland, Auckland, New Zealand; ^5^College of Medicine, University of Leicester, Leicester LE1 7RH, UK

## Abstract

In recent years, the study of metabolomics has begun to receive increasing international attention, especially as it pertains to medical research. This is due in part to the potential for discovery of new biomarkers in the metabolome and to a new understanding of the “exposome”, which refers to the endogenous and exogenous compounds that reflect external exposures. Consequently, metabolomics research into pregnancy-related issues has increased. Biomarkers discovered through metabolomics may shed some light on the etiology of certain pregnancy-related complications and their adverse effects on future maternal health and infant development and improve current clinical management. The discoveries and methods used in these studies will be compiled and summarized within the following paper. A further focus of this paper is the use of hair as a biological sample, which is gaining increasing attention across diverse fields due to its noninvasive sampling method and the metabolome stability. Its significance in exposome studies will be considered in this review, as well as the potential to associate exposures with adverse pregnancy outcomes. Currently, hair has been used in only two metabolomics studies relating to fetal growth restriction (FGR) and gestational diabetes mellitus (GDM).

## 1. Introduction

The speed in which new medical analytical techniques have been developed during the past century has been monumental. “Omics”, a group of biological fields comprising genomics, transcriptomics, proteomics, and metabolomics allow for high-throughput, simultaneous analysis of vast numbers of molecules within a biological sample [1–6]. The last of these, metabolomics, is defined as the study of metabolites, a recognized field of study arising in the 1960s aimed at developing a better understanding of cellular biology [7–9]. However, its nomenclature is relatively new; the term “metabolomics” was coined in the early 2000s [10]. The study of metabolomics facilitates an increased understanding of all the endogenous and exogenous low-molecular weight molecules (<1500 Da) which are downstream products from interactions on a genomic or proteomic level [11–13].

Within metabolomics, the study of the “exposome”, defined as an individual's total exogenous footprint derived from exogenous factors that exert a significant influence on an individual's biochemistry, is a relatively new but quickly popularizing discipline for biomarker screening [14, 15]. The total metabolic profile therefore reflects the biochemical changes of a living cell or organism brought about by certain physiological conditions or in response to an external environmental stimulus. This provides insight into the underlying mechanism of disease development and progression and can act as the bridge between genetic factors and environmental exposures, revealing clues to the origin of the resultant phenotypes of different individuals [16–18].

Metabolomics has become a fast growing area of interest for research into the prognosis, screening, diagnosis, and treatment of many diseases [19–23]. Medical metabolomics research has been focused on the identification and investigation of relevant metabolites formed during certain pathological metabolic reactions. The process begins with untargeted screening of the metabolome (the entire set of small molecules contained in a biological sample). Overall associations between metabolites with significantly altered levels from control are identified and analyzed to generate a hypothesis. A more targeted approach is then utilized, focusing on the metabolites with significantly altered values to further investigate the consistency of results and prove or disprove the proposed hypothesis. A variety of biological specimens, namely, serum, urine, tissue, cerebrospinal fluid, hair, saliva, stool, and exhaled breath, have been used in analysis to study human pathology [13, 24–26].

Mass spectrometry-based metabolomics has been utilized in studies of pregnancy complications such as preeclampsia (PE), fetal growth restriction (FGR), gestational diabetes mellitus (GDM), and preterm birth (PB). The biomarkers related to these complications are analyzed to obtain better knowledge concerning the underlying pathological changes at a cellular metabolic level. The end goal is to develop a more individualised method of screening and treatment plan for each patient according to their metabolic phenotype. However, a greater understanding of the pathophysiology of each condition is first required [27].

In initial metabolomic studies of pregnancy-related complications, urine and blood were used. However, the use of hair as a metabolomic sample has generated increasing interest due to its noninvasive nature and the stability of compounds residing in the hair matrix. Unlike urine and blood, results from a metabolomic investigation of hair do not fluctuate from hour to hour. The metabolomic study of human hair will allow for retrospective studies to be performed, by way of segmental analysis on a single strand of hair [28, 29]. Hence, metabolomic analysis of hair can provide a record of the chemical effects from long-term exposure of known teratogenic pollutants such as tobacco, alcohol, and drugs [30–32].

## 2. Metabolomics of Adverse Pregnancy Outcomes

### 2.1. Preeclampsia

Preeclampsia is prevalent in 3-8% of all pregnancies worldwide [33], with a high maternal mortality rate of >50,000 per year [34]. It is defined by hypertension with a BP of over 140/90 mmHg measured at two consecutive times within a 6-hour period and proteinuria of >300mg/day, in pregnant women with gestational ages beyond 20 weeks. The precise aetiology of preeclampsia remains unclear. However, it is generally accepted that the condition arises from defective vascularization of the placenta. Abnormal extravillous trophoblastic penetration into the muscular layer of spiral arteries in the uterus leads to reduced uteroplacental blood flow. Diminished placental blood perfusion will then give rise to ischaemia, oxidative stress, and inflammation. The two characteristic signs of preeclampsia, namely, proteinuria and hypertension due to endothelial injury, arise from the release of inflammatory cytokines released in the aforementioned process [33].

Preeclampsia can potentially progress to complications such as eclamptic seizures and hemolysis, elevated liver enzymes and lowered platelet count syndrome (HELLP syndrome), and acute complications such as renal or cardiac failure [33, 35, 36]. Long-term maternal implications include an increased risk of developing cardiovascular disease postpartum. Preeclampsia also influences fetal growth and development, often resulting in fetal growth restriction (FGR) and preterm birth (often iatrogenic) [37].

Previous metabolomics investigations are summarized in Table 1, demonstrating the significantly altered values of certain metabolites in patients with preeclampsia as compared to healthy pregnancies. Lipid peroxidation and altered lipoprotein concentrations have been linked to endothelial dysfunction and oxidative stress, and indeed one of the notable findings consistent in several of these studies is the altered values of metabolites related to lipid metabolism. Carnitine, one of the metabolites related to transport of lipids from cytoplasm to mitochondria, has also been reported at significantly altered levels in preeclampsia (Table 1). In addition, difference has been reported in levels of choline, an essential nutrient functioning in the metabolism of phospholipids; choline can also influence inflammation, apoptosis, and angiogenesis [38, 39]. Mukherjee et al. proposed an interesting hypothesis that change of fatty metabolism in the placenta associated with early-onset preeclampsia (<34 weeks of gestation) can be reflected in maternal plasma metabolites [40]. Additionally, changes in the concentration of certain amino acids can be related to the risks for development of preeclampsia, such as a decrease in taurine, an amino acid released during trophoblast invasion into spiral arteries [41, 42].

### 2.2. Fetal Growth Restriction and Small for Gestational Age

A fetus is defined as small for its gestational age (SGA) when its estimated fetal weight falls below the 10th percentile [43, 44]. Evidence of SGA is not enough to determine a pathological diagnosis, as several other physiological factors such as maternal ethnicity and genetics can contribute to a low estimated fetal weight (EFW). A pathologic diagnosis of fetal growth restriction (FGR) (also known as intrauterine growth restriction, IUGR) is more likely when SGA is coupled with abnormal test results (such as an abnormal Uterine Artery Doppler) [43].

FGR can be attributed to several different factors, such as fetal genetic abnormalities and congenital infections (e.g., toxoplasmosis, malaria, and rubella). However, the majority of FGR is associated with some form of placental perfusion insufficiency [45]. Fetuses with FGR are at high risk of perinatal mortality and development of long-term morbidities such as neurodevelopmental delay, diabetes, and adult hypertension and diabetes [46, 47]. Current treatment plans include monitoring, administration of low-dose aspirin, and induction of preterm delivery [44].

Several metabolomic studies of SGA cases have shown significant alterations in relative concentration of certain metabolic classes between affected pregnancies as compared to uncomplicated pregnancies (Table 2). For instance, Horgan et al. (2011) discovered a significant association between altered levels of sphingolipids and phospholipids in maternal blood and SGA [47]. Sphingolipids are a class of fatty acids that act as vital mediators in the cellular processes of apoptosis, proliferation, and stress responses [48, 49]. Therefore, they may be indicative aberrant cellular processes in cytotrophoblasts, resulting in placental insufficiency and FGR [50, 51]. Phospholipids are another class of fatty acids, comprising the major component of cell membranes. An increase in phospholipid levels in maternal serum is also indicative of cellular damage and apoptosis [52].

Van Vliet et al. (2013) studied metabolomic changes in fetal rabbit brain tissue when FGR was induced; 18 metabolites belonging to certain classes (neurotransmitters, amino acids and fatty acids related to energy metabolism, and oxidative stress) were found to have significantly altered values (p< 0.05) [46]. Of particular note are the significantly decreased values of N-acetylaspartylglutamic acid (NAAG) and N-acetylaspartate (NAA), two cerebral-specific metabolites that are indicative of neuronal density and viability, providing insight to the correlation between FGR and fetal neurodevelopmental delay [53]. Vab Vliet et al. also identified a significant reduction of certain amino acid concentrations (asparagine, ornithine, histidine, and l-lysine) and pyroglutamic acid (precursor to glutamate) in fetal rabbit brain with induced FGR [46]. As amino acids and glutamate are important for brain growth, metabolism, and function, these findings are hardly unexpected. However, Favretto et al. (2014) have conversely discovered significantly increased levels of 7 alpha amino acids (including phenylalanine and tryptophan) and glutamate itself in cord blood [54].

### 2.3. Preterm Birth

PB is defined as delivery before 37 weeks of gestation and affects up to 12-13% of pregnancies [55]. It can be further classified into three categories: spontaneous labor with intact membranes (SPB), preterm premature rupture of the membranes (PPROM), and medically induced preterm labor (IPB, including cesarean sections for maternal and fetal indications) [55]. Understanding of the etiological processes that lead to PB remains limited. However, most SPB cases result from one or more of the following pathological processes: amniochorionic decidual or systemic inflammation, activation of the maternal/fetal hypothalamic–pituitary–adrenal, decidual haemorrhage, or pathological distension of the uterus. Cervicovaginal secretions or fluid could be collected easily in a noninvasive and repetitive way, at different times of gestation and parturition. In many cases, abnormal cervical structure, local inflammation, and high cervicovaginal fluid acetate integral of women appear as major precipitating factors of SPB [56, 57].

Although limited metabolomics study has been performed on PB, Maitre and Oladipo noted a heightened concentration of lysine (an essential amino acid) in maternal urine and premature neonatal plasma, respectively (Table 3) [45, 58]. Maitre et al. (2014) have also noted an elevated lysine and steroid conjugate levels and significantly decreased levels of formate in urine samples related to spontaneous preterm birth (SPB). They speculated that an increase in N-acetylglycoprotein fragment in these urine samples was proportionately linked to higher risk of medically induced preterm birth (IPB) [45]. Since N-acetylglycoproteins are known to be inflammation-induced acute phase proteins in serum, this is not an unexpected finding [59]. However, it shows promising potential for early diagnosis of IPB indications.

### 2.4. Gestational Diabetes Mellitus

Gestational diabetes (GDM) is currently defined as any degree of glucose intolerance with onset or first recognition during pregnancy [60]. The diagnostic criteria vary, but are based on fasting plasma glucose values and glucose value after a glucose load, typically tested at weeks 24-28 of pregnancy [61, 62]. GDM has an incidence of up to 15-20%, according to availability of sophisticated screening methods, population demographics, and criteria for diagnosis [60, 61]. Risk factors that have been correlated with GDM include advanced maternal age, race/ethnicity, obesity, and family history of type 2 diabetes mellitus (T2DM) [61].

As shown in Table 4, several metabolites, such as those of lipids, glycerophospholipid, and carnitines, have been noted across various studies of GDM [63–65]. Another class of these metabolites is the branched-chain amino acids (BCAA). BCAAs have proven involvement in insulin resistance and suppression of insulin secretion, by impacting the mitochondria in pancreatic beta cells, and show an association with higher risk of developing GDM [66–69]. Several fatty acids and downstream products of fatty acid metabolism (which are associated with insulin resistance and glucose metabolism) have also shown downregulation in serum from maternal hyperglycemia and GDM cases [70–72]. Dudzik et al. (2014) discovered several significant differences in relative metabolite concentrations in the plasma and urine samples between hyperglycemic and healthy pregnant women. One of these was a significant decrease in glycerophospholipid levels, which could be attributed to change in glucose metabolism due to beta-cell dysfunction in cases of glucose intolerance [65].

Long-term effects on the mother include postpartum T2DM and cardiovascular disease [60, 61]. Furthermore, infants born to women with GDM are at increased risk for respiratory distress syndrome, macrosomia, jaundice, and obesity or other metabolic syndromes in later life [73, 74]. When diagnosed early, these adverse short-term and long-term health issues may be reduced through lifestyle intervention [60]. Therefore, the study and discovery of potential biomarkers for GDM are significant not only for its benefits for diagnosis and treatment of GDM itself, but also for prevention of further morbidities associated with T2DM.

## 3. Hair Metabolomics

### 3.1. General Use of Hair in Clinical Applications

The use of hair as a biological specimen is commonplace in the measurement of toxic exposures. It began with accurate estimation of exposure to heavy metals and alcohol in the 1960s. Hair specimen have been used to determine the level of exposure to smoking, pesticides and drugs [7, 75, 76]. All of these substances lead directly or indirectly to adverse pregnancy outcomes [77–84]. Chemical markers detected in human hair specimens have proven to be both objective and accurate in the measurement of environmental exposure. One advantage of using hair as a biological sample is that it provides a broader view of an individual's metabolic profile. Both endogenous compounds and environmental chemicals are assimilated into hair during growth. Therefore, hair provides a metabolite profile that reflects the long-term effect of environmental exposure on pregnancy outcomes. In contrast, the more conventional samples such as blood and urine only provide transient biochemical profiles.

In addition, hair is a stable biological sample and can be stored for longer periods of time; no special preparations need to be conducted to prevent sample degradation, thus simplifying transport between laboratories.

There are, however, several limitations related to the use of hair in metabolomic studies. Lower concentrations of toxic substances are incorporated into hair as compared to those that can be analyzed in blood and urine samples. Therefore, instruments with higher sensitivity are required [85, 86]. Furthermore, gender, nutrition, melanin content of hair, lipophilicity, pH of molecules, agricultural exposures, and external contamination (through sweat or sebum) may all affect the incorporation of trace elements into hair [87–90]. Certain hair treatments, even as simple as shampoo, also affect the concentration of analytes found in hair.

#### 3.1.1. Trace Element Exposure

Humans are commonly exposed to a variety of toxic trace elements. Arsenic, cadmium, lead, and mercury are known teratogenic factors and can contribute to neurotoxicity during pregnancy [82, 91–96]. While the more conventional samples such as urine and blood provide a more accurate measurement of physiological toxin concentrations, hair can be used to conduct a retrospective study that could provide more information of the times the toxin was ingested and the accumulation of toxins in the body over time.

Analytical methods have been dramatically improved in recent decades and hybrid generation-atomic fluorescence spectrometry [97, 98], inductively coupled plasma-mass spectrometry (ICP-MS) [87, 99], and atomic absorption spectrometry [88, 100, 101] are utilized to analyze trace elements in hair samples. A more simplistic method for ICP-MS was further developed to cut down on time and resources by coupling high-performance liquid chromatography with ICP-MS [102]. Another method, laser ablation-ICP-MS, gives the spatial distribution of trace elements in a single strand, thereby giving a timeline for exposure with a high degree of accuracy from a minute specimen [103–105].

#### 3.1.2. Drug Abuse Analysis

Measuring complex molecules such as drugs in hair has proved to be more challenging than analysis of trace elements. While trace elements remain unmodified, drugs can be catabolised and it is often the byproducts or downstream metabolites that are incorporated. Therefore, analyses of these compounds require more sophisticated analysis techniques such as GC-MS and LC-MS. Nevertheless, analysis of hair samples remains a method for determining the presence or absence of certain drugs and to obtain a better understanding of absorption and metabolism of different drugs [106].

There are several advantages to using hair samples in investigations of illicit drug exposure. Firstly, it can be collected in person, thus removing the possibility of substitution, which is a common problem in urine sampling [107]. Secondly, unlike the fast clearance rate of these compounds (e.g., heroin) in blood and urine, drugs, and their byproducts remain stable in hair [106, 108, 109]. Finally, segmental analysis of hair samples allows one sample to give a picture for months compared to multiple blood tests, which could be useful for a longitudinal monitor of drugs abstinence [110].

Exposures to certain illicit and pharmaceutical drug compounds such as ACE-inhibitors and heroin during pregnancy have been proven to exert a teratogenic effect, providing a further indication in pregnancy [111–113].

The usual limitations of metabolomics hair analysis should be kept in mind. Moreover, Mercolini L. et al. discovered potential bias from external contamination of hair, for example, that consumption of cannabis could only be confirmed when both THC (tetrahydrocannabinol) and its metabolite THC-COOH were detected in the hair samples [114].

#### 3.1.3. Alcohol Consumption via Hair Analysis

During the last decade, monitoring of alcohol consumption has advanced with the discovery of two types of alcohol metabolite, ethyl glucuronide (EtG), and fatty acid ethyl esters (FAEEs) (ethyl myristate, ethyl palmitate, ethyl oleate, and ethyl stearate) in hair [115–117]. Sensitivity and specificity of GC-MS and LC-MS analysis for detection and measurement of these compounds in hair are excellent and are often utilized in traffic law enforcement and liver transplant allocation [118–121]. Originally, it was believed that FAEE or EtG in hair can only be used for identifying rather than quantifying alcohol consumption [122]. However, in 2016 the Society of Hair Testing published a consensus for workable cut-off values (Table 5). Cut-off values for FAEEs are adjusted according to hair segment used, to account for possible external concentrations of FAEEs into hair from cosmetic treatments.

EtG is predominantly incorporated into hair matrix by sweat [123], while FAEE incorporation is by sebum [124]. Tsanaclis et al. (2009) and Crunelle et al. (2015) discovered that one of the drawbacks of using only EtG in hair for measurement of alcohol consumption is that normal hair hygiene practices (e.g., shampooing) and cosmetic treatments (dyeing and bleaching) greatly reduces the concentration of EtG in hair as it is a hydrophilic compound [125, 126]. However, EtG concentration in hair is not influenced by the use of alcohol-containing hair products or by the use of hair spray, wax, gel, oil, or grease [127]. In contrast, FAEE is virtually unaffected by shampoo washing or dyeing, but its concentration may be increased by ethanol-containing lotions and hairspray [127, 128]. All of these factors and more (such as the site of hair collection) must be considered when determining an individual's abstinence or alcohol dependence. EtG and FAEEs are used in combination to increase reliability of the results [129].

#### 3.1.4. Evaluating Tobacco Smoke Exposure Via Hair Analysis

During the past century, a large number of studies have been conducted on the long-term and short-term teratogenic effects of cigarette smoke [75, 130]. Nicotine and its catabolite cotinine are used in tobacco smoke exposure measurement. However, due to the short half-life of nicotine and cotinine, analytical techniques using conventional samples such as blood [131] and urine [132] have yet been able to accurately quantify the harmful concentrations and level of risk in development of pregnancy complications for pregnant women and their fetuses [131, 132].

Analysis of nicotine and cotinine in hair has been heralded as a solution. Several analytical techniques, namely, GC [75], RPHPLC [133], GC-MS [130], and HPLC [134] have been used to identify smokers. Of particular interest is the GC-MS platform and its ability to evaluate levels of passive smoking by hair sample [135], as it is less prone to interference and a smaller sample size is needed for analysis (e.g., 5 mg of hair from one test subject) [135]. Exposure to polycyclic Aromatic Hydrocarbons (PAHs), another substance found in cigarette smoke and heavy industry, can also be quantified in hair through GC-MS [130].

#### 3.1.5. Evaluating Pesticide Exposure via Hair Analysis

In recent decades, many pesticides have been outright banned or its use severely restricted in Europe and several other countries [136]. However, pesticide use is still widespread, despite well-known carcinogenic, mutagenic, and teratogenic effects [137–141].

Blood and urine are routinely used to assess pesticide intake. These compounds can be directly measured in blood while their catabolites can be analyzed in urine. In addition to providing a method for retrospective analysis of pollutant exposure, both the compound itself and its catabolites can be analyzed in a single hair sample. GC-MS [142] or GC-MS/MS [143] are validated methods but both require a precleanup procedure, either liquid-liquid extraction (LLE) or solid-phase extraction (SPE), to purify the analytes and enhance sensitivity. A study conducted by Duca et al. (2014) found that SPE gives a better recovery rate of the analytes compared to LLE and reduces the background noise of the analysis via detecting sixty-seven pesticides and metabolites from different chemical classes including organochlorines, organophosphates, carbamates, pyrethroids, ureas, azoles, phenylpyrazoles, and neonicotinoids. [144]. In 2012, Salquèbre et al. applied an improved clean-up procedure developed from SPE called Solid-phase microextraction (SPME) that in combination with gas chromatography tandem (triple quadrupole) mass spectrometry was able to analyze smaller sample amounts due to its higher sensitivity [85].

## 4. Hair Metabolome Related to Adverse Pregnancy Outcomes

The application and advantages of hair as a biological specimen have previously been discussed in the context of biological screening and toxin exposure. The use of hair to investigate the metabolome of pregnant women and research adverse pregnancy outcomes holds great potential. The incidence of adverse pregnancy outcomes is influenced by environmental and physiological changes from preconception until delivery. Analysis of hair could provide a wide perspective of the whole process as both endogenous (metabolome) and exogenous compounds (exposome) are incorporated. However, thus far, this potential has not been fully realized. There have only been two studies investigating the hair metabolome in pregnant women who suffer from certain pregnancy-related complications. These studies were done by Sulek et al. and He et al. on FGR [145] and GDM [146], respectively (Table 6).

In the FGR study, hair samples were taken from 83 women at 26-28 weeks of gestation. 41 of these women subsequently delivered SGA fetuses, and the 42 remaining women delivered fetuses of normal weight, serving as controls. GC-MS based metabolite profiling of the hair samples was used to discover significant variations in a range of metabolites between cases and controls including amino acids, amino acid derivatives, fatty acids, cofactors, and antioxidants. Based on these results, a biomarker model was capable of distinguishing pregnancies complicated by FGR from normal pregnancies, using five metabolites, with a receiver-operating curve of 0.99. The authors also reported increased levels of heptadecane in samples obtained from FGR pregnancies. Heptadecane is an exogenous alkane hydrocarbon incorporated into the metabolome through air pollution and/or food contamination and may represent an environmental trigger that leads to FGR. Furthermore, elevated levels of NADPH/NADP in combination with decreased levels of glutathione observed in the FGR cases suggest redox imbalance and placental oxidative stress that may be another precipitating factor of FGR [46, 145].

More recently, the GDM study conducted by He et al. (2015) studied hair metabolite profiles from 47 cases diagnosed with GDM as compared to 47 controls with no pregnancy complications, at around 24-28 weeks of gestation. The hair metabolome analysis of GDM identified a significantly elevated level of adipic acid, a compound that had been found at elevated levels in urine of subjects with Type II diabetes and associated with oxidative stress [146–149]. One limitation of this study, however, was that hair analysis was performed on the whole hair strand. Therefore, the results represent the metabolic profile of both preconception and early gestational periods [146].

Both studies thus show promising results that could advance the understanding and clinical approach to these pregnancy complications.

## 5. Relating the Exposome to Adverse Pregnancy Outcomes

The term “exposome” was first defined by Christopher Wild in 2005 and then underwent several modifications as the understanding of it developed over time [150]. The latest definition of the exposome, suggested by Miller and Jones, is “the cumulative measure of environmental influences and associated biological responses throughout the lifespan including exposures from the environment, diet, behaviour, and endogenous processes” [14]. Evaluating adverse pregnancy outcomes to pollutants, drugs, and various exposures based on maternal metabolite profiles seem to be a logical next step. Exposome markers not only give rise to specific phenotypes but also modify the metabolic phenotype depending on the health status of the individual. In addition to external exposome, the endogenous exposome, which are the chemicals generated within the human body in response to external stimuli through various physiological processes (e.g., oxidative stress, inflammation, or infections) should also be investigated [151].

Currently, the major focus in pregnancy-related exposure investigations is on pesticides, tobacco smoke, drug abuse, alcohol dependence, and exposure to mercury [79, 152, 153]. These pollutants can be transported from mother to fetus through the placenta. Prenatal and neonatal samples such as maternal and infant hair, meconium, maternal blood, and cord blood have been used in studies done on fetal and maternal exposures. Results from these studies show that meconium provides the most sensitive data representing* in utero* fetal exposure, while maternal hair sampling is the most stable for detecting the maternal exposome [32, 154–156].

Fetal exposure to mercury and more specifically methylmercury (a neurotoxin) during pregnancy could occur through maternal consumption of mercury-contaminated fish [157, 158]. Fetal exposure to pesticides could occur through contaminated food and water, inhalation of pesticide-polluted air, or skin contact. Some pesticides are also neurotoxic and could result in mental retardation, learning disabilities, autism, attention deficit-hyperactivity disorders, and deficiencies in motor development in the infant [159, 160]. Furthermore, increased risks of IUGR, PTB, miscarriage, and fetal alcohol syndrome have been associated with exposure to pollutants during pregnancy [161–163]. One study by Bonvallot et al. (2013) has demonstrated that 5 metabolites (glycine, threonine, lactate, glycerophosphocholine, and citrate) in the urine were significantly different between pesticide-exposed and unexposed pregnant women [164]. Comprehending the associations between the exposome and adverse pregnancy outcomes and future health of fetus is crucial in risk estimation and prediction of developing pregnancy complications, and further studies into the exposome should be conducted.

## 6. Conclusion

It is well-established that the first 1000 days of human life, calculated from conception, are crucial in determining well-being throughout the entire lifespan [165]. The most recent research studies have further suggested that maternal health condition even before conception is also involved in determining fetal health [166]. Therefore, the maternal metabolome should be monitored or assessed as early as possible. Given that many pregnancies are unplanned and first blood draw or urine collection is done in the pregnancy clinic, a biological sample that could be collected after conception but able to reveal the preconception metabolome would prove to be extremely valuable. This is one main advantage of using hair as a biological sample. In addition, analysis of the hair metabolome also holds potential for early diagnosis and risk identification biomarkers for various pregnancy complications and harmful exposures during pregnancy. Preventative plans and /or personalized treatment plans could be created from the data obtained from these studies. Other advantages of using hair as a biological sample are that hair sampling is noninvasive and safe for both mother and fetus, and the hair metabolome is stable and can be preserved for months at room temperature without any special preparation. Thus, further studies of the hair metabolome relating to pregnancy complications could open the door to many significant practical advances in the way these conditions are dealt with in the clinical setting.

## Figures and Tables

**Table 1:** Examples of metabolomics studies associated with preeclampsia (EO-PE: early-onset preeclampsia, LO-EP: late-onset preeclampsia, and PE: preeclampsia).

Sample specimen	Participants (n)	Outcomes	Analytical platforms	Metabolites	Statistical analysis	References
Serum	80 (20 EO-PE, 20 LO-PE)	EO-PE, LO-PE, controls for both	FTIR spectroscopy, H^1^ NMR	FTIR results: carbohydrate, protein and lipid region sign. Different in EO-PE, H^1^ NMR model included: ↑Glutamate, choline, alanine, lactate ↓ arginine, citrate	P<0.001, 95% CI	[40]
Plasma	Cohort 1: 40 (20 PE); cohort 2: 174 (87 PE) [167]	Preeclampsia and controls	UPLC-LTQ Orbitrap-MS	Alanine, 2-Hydroxy-3-methyl-butanoic acid, 2-Ethyl-3-hydroxypropionic acid, 2-Oxoglutaric acid, Glutamic acid, Xylitol or ribitol, Uric acid, Creatinine	P<0.01	[168]
Plasma (15+/- 1 weeks of gestation)	120 (discovery, 60 PE)	PE and controls	UPLC-MS	Study 1: 45 unique metabolites divided into 11 clear metabolite classes: amino acids, carbohydrates, carnitines, Eicosanoids, fatty acids, keto or hydroxy acids, lipids, phospholipids, porphyrins, phosphatidylserine, and steroids.	study 1: P<0.05	[169]
	79 (validation, 39 PE)			Study 2: data mining and modeling techniques it gave rise of a model containing 14 metabolites (5-Hydroxytryptophan, Monosaccharide(s), Decanoylcarnitine, Methylglutaric acid and/or adipic acid*∗*, Oleic acid, Docosahexaenoic acid and/or Docosatetraenoic acid, -Butyrolactone and/or oxolan-3-one, 2-Oxovaleric acid and/or oxo-methylbutanoic acid, Acetoacetic acid, Hexadecenoyl- eicosatetraenoyl-sn- glycerol*∗*, Di-(octadecadienoyl)-sn- glycerol*∗*, Sphingosine 1-phosphate, Sphinganine 1-phosphate, Vitamin D3 derivatives) to be a robust model to predict PE with AUC of >0.9	Study 2: P<0.05, robust predictive model of 14 metabolites: AUC of >0.9	
Placenta (first trimester)	Study 1: 12 (terminated pregnancy); study 2: 17 (6 PE)	Late PE, controls	GC-TOF-MS, UPLC-LTQ Orbitrap-MS	classes which are significantly different between term PE and normal term pregnancies: acyl glycerides, phospholipids, fatty acids and related metabolites, amino acids related metabolites, vitamin D-related metabolites, isoprenoids, and steroids	P<0.05	[170]
Serum (11(+0)-13(+6) weeks of gestation)	119 (30 LO-PE, 30 EO-PE)	LO-PE, EO-PE, controls	NMR	1st analysis (late onset PE vs controls): 17 metabolites significant different, of which Glycerol, carnitine, methylhistidine, acetone most important to discriminate based on VIP. 2nd analysis (Late onset vs early onset PE): glycerol, acetate, trimethylamine and succinate most important to discriminate based on VIP analysis	P<0.05, complex model (metabolites/maternal demographic info): 76.6% sensitivity at 100% specificity, simplified model: 60% sensitivity at 96.6% specificity	[171]
Plasma (11-13 weeks)	90 (30 EO-PE)	EO-PE, controls	NMR	Model 1: metabolites (glutamine, pyruvate, propylene glycol, trimethylamine, hydroxybutyrate) in combination with maternal characteristics (weight and medical disorder) and Model 2: metabolites (glutamine, pyruvate, propylene glycol, trimethylamine, hydroxybutyrate, carnitine, hydroxy isovalerate) in combination with uterine artery PI.	P<0.005, model 1: estimated detection rate is 75.9%, model 2: estimated detection rate is 82.6%	[172]
Serum (11(+0)-13(+6) weeks of gestation)	Discovery: 95 (30 EO-PE); Validation: 63 (20 EO-PE)	EO-PE, controls	NMR	Metabolite-only model: glycerol, 3-hydroxyisovalerate, 2-hydroxybutyrate, acetone, and citrate; combined logistic regression model: glycerol, 3-hydroxyisovalerate, arginine, and UtPI data	Metabolite only model: O.835 of AUC; combined logistic regression model (metabolite plus uterine Doppler pulsatility index): 0.916 of AUC for early PE detection in validation group	[173]
Serum (11-14 weeks of gestation)	82 (41 PE)	PE and controls	LC-MS/MS	Hydroxyhexanoylcarnitine, phenylalanine, glutamate, alanine, were significantly higher in PE cases compared to controls and adjusted by BMI, ethnicity and pregestational diabetes,	P<0.05, individual metabolites AUC of 0.77-0.80, combined metabolites AUC of 0.82 for all PE cases and 0.85 for EO-PE cases	[174]
Urine, Serum at time of diagnosis	30 (10 PE, 10 controls, 10 non-pregnant)	PE and controls (pregnant and non-pregnant)	NMR	Urine: by PLS-DA: ↑choline and creatinine level, ↓ glycine levels in PE aces vs healthy pregnancies, women with early onset PE had ↑Trimethylamine-N-oxide, creatinine, ↓choline and creatinine compared to late onset PE; Serum: lipid content PE cases>normal pregnancies>nonpregnant women. Distribution of lipoproteins was also different between groups with PE cases most ↑ levels from VLDL and LDL and ↓ levels of HDL, PE cases had significantly ↓ levels of histidine compared to healthy pregnancy.	P<0.05 (PCA) and P<0.001 (PLS-DA), 95% significance of the predictive model	[175]
Urine, serum (first trimester)	599 (26 PE, 21 gest. Hypertension)	PE, gestational hypertension, controls	NMR	↑ levels of creatinine, glycine, 4-deoxythreonic acid, *α*-hydroxyisobutyrate, histidine and dimethylamine, ↓ levels of hippurate, lactate and proline betaine in the urine of women which developed preeclampsia. Women who developed gestational hypertension had an additional ↓ of citrate level in urine. In serum samples: ↑ lipid levels in both hypertensive groups. ↓ levels of phosphatidylcholines, glucose, lactate, and alanine.	P<0.05,Urine: 51.3% sensitivity for PE and 40% gestational hypertension, Serum: 15% sensitivity for PE and 33% gestational hypertension	[176]
Serum(8(+0)-13(+6) weeks of gestation)	667 (68 EO-PE, 99 LO-PE)	EO-PE, LO-PE, controls	UPLC-MS/MS, LC-MS	EO-PE vs controls: taurine and asparagine; LO-PE vs controls: glycylglycine		[41]

**Table 2:** Examples of metabolomic studies associated with FGR/SGA.

Sample specimen	Participants (n)	Outcomes	Analytical platforms	Metabolites	Statistical analysis	References
Cord blood: serum	43 (22 IUGR)	IUGR, AGA (appropriate for gest. Age)	LC-HRMS (orbitrap)	Phenylalanine, tryptophan, and glutamate to discriminate between IUGR and AGA	P<0.05, ROC curve: sensitivity between 91-100% and specificity between 85-89%	[54]
Fetal rabbit brain tissue	Living: 9 (4IUGR) Stillborn: 7 (6 IUGR)	IUGR, control	LC-QTOF-MS	N-acetylaspartylglutamic acid (NAAG), N-acetylaspartate (NAA), ornithine, L-lysine, asparagine, histidine, and leucine intermediate 2-keto-isovalerate, succinate, pantothenate, malondialdehyde and 3-nitrotyrosine, purine, 3,4-dihydroxybutyric acid, nucleotide GMP, docosahexaenoic acid (DHA), palmitoleic acid and oleic acid	P<0.05	[46]
Urine (end of the first trimester)	464 (36 FGR, 19 SGA)	FGR, SGA, Controls	NMR	significant association FGR with decreased levels of acetate, formate, tyrosine and trimethylamine in urine adjusted for education, maternal age, parity, smoking during pregnancy; SGA was associated with leucine and N-acetyl neuraminic acid	95% confi.int., AUC for FGR: 63.7-66.3%	[45]
Urine of IUGR newborns	56 (26 IUGR)	IUGR, controls	NMR	Discrimination between IUGR and control neonates by myo-inositol, sarcosine, creatine and creatinine	/	[177]
(a) cord plasma (b) early pregnancy maternal plasma	(a) 14 (8 SGA) (b) 340 (73 SGA)	SGA, control	UPLC-MS	Pregnanediol-3-glucuronide OR 3alpha,20alpha-dihydroxy-5beta-pregnane 3-glucuronide, LysoPC(16:1) OR Cervonyl carnitine,6-hydroxysphingosine OR (4OH,8Z,t18:1) OR 3b-Allotetrahydrocortisol OR 15-methyl-15-PGD2 OR 15R-PGE2 methyl ester, Leucyl-leucyl-norleucine OR Sphingosine 1-phosphate, Cervonyl carnitine AND/OR 1R,25-dihydroxy-18-oxocholecalciferol, 17-[(Benzylamino)methyl]estra-1,3,5(10)-triene-3,17beta-diol, PC, phosphocholine; PGD, Prostaglandin D; PGE, prostaglandin E. significant different between SGA and controls in both studies associated with cord plasma and week 15 plasma	P<0.05, robust predictive model of 19 metabolites of presymptomatic SGA: AUC of 0.9	[47]

**Table 3:** Examples of metabolomics studies associated with preterm birth.

Sample specimen	Participants (n)	Outcomes	Analytical platforms	Metabolites	Statistical analysis	References
Urine (end of the first trimester)	464 (88 spont. PB, 26 ind. PB)	Preterm birth (spontaneous and induced), control	NMR	Spontaneous PB associated with increased urine lysine, clinically induced PB was associated with in overweight and obese women and increased resonance in a N-acetyl glycoprotein	95% confi.int., AUC for SPB: 58.8-59.4%, for IPB: 66%	[45]
Cervicovaginal fluid	219	Preterm birth, controls	NMR	Higher lactate level in term ALR group compared to term and preterm AHR women, acetate was increased in preterm compared to the term group for SYM and AHR group	P<0.05, acetate integrals in PTB versus term for AHR and SYM group: predictive of preterm birth <37 gestational weeks is AUC of 0.78, acetate integrals in PTB versus term Sym group: delivery within 2 weeks of index assessment AUC of 0.84	[57]
Cervicovaginal secretions	15 (5 Spont. PB)	Preterm birth, control	UPLC-QTOF-MS	17 markers were observed to distinguish between preterm birth and control groups; further research is needed	P<0.05	[56]

**Table 4:** Examples of metabolomic studies associated with gestational diabetes mellitus.

Sample specimen	Participants (n)	Outcomes	Analytical platforms	Metabolites	Statistical analysis	References
Plasma	31 (18 GDM)	GDM vs controls	LC-ESI-QqQ-MS/MS	L-asparagine (Asn), L-valine (Val), and L-ornithine (Orn) were decreased and L-citrulline (Cit) was elevated in GDM cases compared to controls	P<0.05	[178]
Serum (3rd trimester)	22 (12 GDM)	GDM vs controls	UPLC-QTOF-MS	9 metabolites were observed with AUC>0.7 to diagnose GDM from healthy controls which are 1- methyladenosine, glucosamine, L-tyrosine, phosphorylcholine, L-lactic acid, 3-methylthiopropionic acid, lysoPC(16:1), L-2- hydroxyglutaric acid, and trans-3-octenedioic acid	P<0.05	[64]
Serum (16 weeks of gestation)	358 (178 GDM)	GDM vs controls	GC-MS	17 metabolites: linoleic acid, oleic acid, myristic acid, d-galactose, d-sorbitol, o-phosphoco- lamine, l-alanine, l-valine, 5-hydroxy-l-tryptophan, l-phenylalanine-phenyl, l-serine, sarcosine, l-pyroglutamic acid, and l-mimosine, l-lactic acid, glycolic acid, fumaric acid, and urea expressed differentiation between GDM cases and controls	Regression analysis, these metabolites together with GDM risk factors (maternal age, family history, prepregnancy BMI, ferritin, CRP, hep- cidin, and total vitamin D) gave rise to an AUC of 0.87	[179]
Plasma (24-27 weeks of gestation, at OGTT test)	24 (9 GDM)	GDM vs controls at OGTT test	FIA-MS/MS and LC-MS for amino acids, acylcarnitines, sphingomyelins, phosphatidylcholines, hexose (glucose) and biogenic amines; Fatty acids analysis by GC-MS	reference model complemented by a clinical model(BMI, AUC of glucose and insulin) gave rise to 8 metabolites C18:0 carnitine, PC aa C34:4, PC aa C36:4, PC aa C38:5, PC ae C36:4, PC ae C36:5, LPC C20:4 and arachidonic acid which showed differences between GDM cases and controls	P<0.01, reference model: 96.4% AUC and clinical model: 99.3% AUC	[63]
Serum (fasting)	192 (96 GDM)	GDM vs controls	LC-MS	anthranilic acid, alanine, glutamate, allantoin and serine were increased and creatinine decreased in GDM cases compared to controls	P<0.05	[180]
Serum (20 weeks)	48 (22 GDM)	GDM vs controls	GC-MS	Itaconic acid with P=0.0003 was significantly increased in women who developed GDM later on during pregnancy compared to controls, cis-aconitate levels were also higher in GDM cases and verging on statistical difference (P=0.013)	P<0.01	[74]
Urine and plasma (Fasting)	40 (20 GDM)	GDM vs controls	LC-QTOF-MS(plasma), GC-Q/MS(plasma), CE-TOF/MS(urine)	After ROC analysis metabolites with a ROC area >0.94 and has shown a discriminative ability by 25 lysoglycerophospholipids, arachidonic 20:4) and docosahexaenoic (22:6) acid methyl esters, and taurine-conjugated bile acids. Lipoxin was another lipid which showed a high discriminative power and associated with diabetic outcome	ROC area>0.94	[65]
Urine (8-20 gest. week, week 28+/-2, 10-16 weeks after pregnancy)	609 (13% GDM)	GDM vs controls	NMR	Significant increase of citrate levels associated with GDM severity	P<0.05, R2>95%	[181]

**Table 5:** Consensus on alcohol markers in hair analysis by Society of Hair Testing.

	Total Abstinence	Chronic Excessive Consumption	Hair segment
EtG	<7 pg/mg	≥30 pg/mg	proximal scalp hair segment up to 6 cm
FAEEs	<0.12ng/mg	≥0.35 ng/mg	0-3 cm proximal scalp hair segment
	<0.15ng/mg	≥0.45 ng/mg	0-6 cm proximal scalp hair segment

**Table 6:** Examples of hair metabolomic studies associated with complicated pregnancies.

Sample specimen	Participants (n)	Outcomes	Analytical platforms	Metabolites	Statistical analysis	References
Maternal hair	83(41 FGR,42 Controls)	FGR vs Controls	GC-MS	5 discriminating metabolites (lactate, levulinate, 2-methyloctadecanate, tyrosine, and margarate) were observer between FGR and normal pregnancies	P<0.01	[145]
Maternal hair	94(47 GDM,47 Controls)	GDM vs controls	GC-MS	adipic acid significantly elevated in GDM compared to the control group (P=0.002)	P<0.05	[146]

## Abbreviations

MDPI: Multidisciplinary Digital Publishing Institute
DOAJ: Directory of open-access journals
FGR: Fetal Growth Restriction
GDM: Gestational diabetes mellitus
PE: Preeclampsia
PB: Preterm birth
HELLP: Hemolysis, elevated liver enzymes, and lowered platelet count syndrome
EO-PE: Early-onset preeclampsia
LO-PE: Late-onset preeclampsia
FTIR: Fourier transform infrared spectroscopy
H1-NMR: Proton nuclear magnetic resonance spectroscopy
UPLC-LTQ Orbitrap-MS: Ultra-Performance Liquid Chromatography-Linear Orbitrap-Mass Spectrometry
GC-TOF-MS: Gas Chromatography-Time of Flight-Mass Spectroscopy
AUC: Area under curve
IUGR: Intrauterine growth restriction
EFW: Estimated fetal weight
SGA: Small for gestational age
NAAG: N-acetylaspartylglutamic acid
NAA: N-acetylaspartate
AGA: Appropriate for gestational age
LC-HRMS: Liquid Chromatography-High Resolution Mass Spectrometry
SPB: Spontaneous preterm birth
PPROM: Preterm premature rupture of the membranes
IPB: Induced preterm birth
T2DM: Type 2 diabetes mellitus
BCAA: Branched-chain amino acid
LC-ESI-QqQ-MS/MS: Liquid Chromatography-Electrospray Ionization-triple quadrupole- tandem Mass Spectrometry
ICP-MS: Inductively Coupled Plasma-Mass Spectrometry
THC: Tetrahydrocannabinol
THC-COOH: 1-nor-9-carboxy-delta-9-tetrahydrocannabinol
LLE: Liquid-liquid extraction
SPE: Solid-phase extraction.

## References

[1] Markley J. L., Brüschweiler R., Edison A. S. (2017). The future of NMR-based metabolomics. *Current Opinion in Biotechnology*.

[2] Vinaixa M., Schymanski E. L., Neumann S., Navarro M., Salek R. M., Yanes O. (2016). Mass spectral databases for LC/MS- and GC/MS-based metabolomics: state of the field and future prospects. *TrAC Trends in Analytical Chemistry*.

[3] Boersema P. J., Raijmakers R., Lemeer S., Mohammed S., Heck A. J. R. (2009). Multiplex peptide stable isotope dimethyl labeling for quantitative proteomics. *Nature Protocols*.

[4] Thompson A., Schäfer J., Kuhn K. (2003). Tandem mass tags: a novel quantification strategy for comparative analysis of complex protein mixtures by MS/MS. *Analytical Chemistry*.

[5] Wang Z., Gerstein M., Snyder M. (2009). RNA-Seq: a revolutionary tool for transcriptomics. *Nature Reviews Genetics*.

[6] Kumar G., Kocour M. (2017). Applications of next-generation sequencing in fisheries research: A review. *Fisheries Research*.

[7] Jaakonmaki P. I., Knox K. L., Horning E. C., Horning M. G. (1967). The characterization by gas-liquid chromatography of ethyl *β*-D-glucosiduronic acid as a metabolite of ethanol in rat and man. *European Journal of Pharmacology*.

[8] Horning M. G., Boucher E. A., Stafford M., Horning E. C. (1972). A rapid procedure for the isolation of drugs and drug metabolites from plasma. *Clinica Chimica Acta*.

[9] Horning E. C., Horning M. G. (1971). Metabolic profiles: gas-phase methods for analysis of metabolites.. *Clinical Chemistry*.

[10] Fiehn O., Kopka J., Dörmann P., Altmann T., Trethewey R. N., Willmitzer L. (2000). Metabolite profiling for plant functional genomics. *Nature Biotechnology*.

[11] Wishart D. S. (2007). Current progress in computational metabolomics. *Briefings in Bioinformatics*.

[12] Hollywood K., Brison D. R., Goodacre R. (2006). Metabolomics: current technologies and future trends. *Proteomics*.

[13] Bouatra S., Aziat F., Mandal R. (2013). The human urine metabolome. *PLoS ONE*.

[14] Miller G. W. (2014). The Exposome: Purpose, Definition, and Scope. *The Exposome*.

[15] Miller G. W. (2014). The exposome in environmental health sciences and related disciplines. *The Exposome*.

[16] Bundy J. G., Davey M. P., Viant M. R. (2009). Environmental metabolomics: A critical review and future perspectives. *Metabolomics*.

[17] Viant M. R. (2008). Recent developments in environmental metabolomics. *Molecular BioSystems*.

[18] Montoliu I., Genick U., Ledda M. (2013). Current status on genome-metabolome-wide associations: An opportunity in nutrition research. *Genes & Nutrition*.

[19] Zhao Y.-Y., Cheng X.-L., Vaziri N. D., Liu S., Lin R.-C. (2015). UPLC-based metabonomic applications for discovering biomarkers of diseases in clinical chemistry. *Clinical Biochemistry*.

[20] Xia J., Broadhurst D. I., Wilson M., Wishart D. S. (2013). Translational biomarker discovery in clinical metabolomics: An introductory tutorial. *Metabolomics*.

[21] Sébédio J. L., Polakof S., Brennan J.-L., Sébédio L. (2015). Using metabolomics to identify biomarkers for metabolic diseases: analytical methods and applications A2. *Metabolomics as a Tool in Nutrition Research*.

[22] Brindle J. T., Antti H., Holmes E. (2003). Erratum: Rapid and noninvasive diagnosis of the presence and severity of coronary heart disease using 1H-NMR-based metabonomics (Nature Medicine (2002) 8 (1439-1445)). *Nature Medicine*.

[23] Mendrick D. L., Schnackenberg L. (2009). Genomic and metabolomic advances in the identification of disease and adverse event biomarkers. *Biomarkers in Medicine*.

[24] Wishart D. S., Knox C., Guo A. C. (2009). HMDB: a knowledgebase for the human metabolome. *Nucleic Acids Research*.

[25] Psychogios N., Hau D. D., Peng J. (2011). The human serum metabolome. *PLoS ONE*.

[26] Wishart D. S., Lewis M. J., Morrissey J. A. (2008). The human cerebrospinal fluid metabolome. *Journal of Chromatography A*.

[27] Holmes E., Wilson I. D., Nicholson J. K. (2008). Metabolic phenotyping in health and disease. *Cell*.

[28] Gizlenti S., Ekmekci T. R. (2014). The changes in the hair cycle during gestation and the post-partum period. *Journal of the European Academy of Dermatology and Venereology*.

[29] Buffoli B., Rinaldi F., Labanca M. (2014). The human hair: From anatomy to physiology. *International Journal of Dermatology*.

[30] Joya X., Mazarico E., Ramis J. (2016). Segmental hair analysis to assess effectiveness of single-session motivational intervention to stop ethanol use during pregnancy. *Drug and Alcohol Dependence*.

[31] Joya X., Marchei E., Salat-Batlle J. (2016). Drugs of abuse in maternal hair and paired neonatal meconium: an objective assessment of foetal exposure to gestational consumption. *Drug Testing and Analysis*.

[32] Ostrea E. M., Bielawski D. M., Posecion N. C. (2009). Combined analysis of prenatal (maternal hair and blood) and neonatal (infant hair, cord blood and meconium) matrices to detect fetal exposure to environmental pesticides. *Environmental Research*.

[33] Anderson U. D., Olsson M. G., Kristensen K. H., Åkerström B., Hansson S. R. (2012). Review: biochemical markers to predict preeclampsia. *Placenta*.

[34] WHO (2005). *The World Health Report 2005-Make Every Mother and Child Count*.

[35] Villar J., Carroli G., Wojdyla D. (2006). Preeclampsia, gestational hypertension and intrauterine growth restriction, related or independent conditions?. *American Journal of Obstetrics & Gynecology*.

[36] Carty D. M., Delles C., Dominiczak A. F. (2008). Novel biomarkers for predicting preeclampsia. *Trends in Cardiovascular Medicine*.

[37] Bellamy L., Casas J.-P., Hingorani A. D., Williams D. J. (2007). Pre-eclampsia and risk of cardiovascular disease and cancer in later life: systematic review and meta-analysis. *British Medical Journal*.

[38] Jiang X., Bar H. Y., Yan J. (2013). A higher maternal choline intake among third-trimester pregnant women lowers placental and circulating concentrations of the antiangiogenic factor fms-like tyrosine kinase-1 (sFLT1). *The FASEB Journal*.

[39] Friesen R. W., Novak E. M., Hasman D., Innis S. M. (2007). Relationship of dimethylglycine, choline, and betaine with oxoproline in plasma of pregnant women and their newborn infants. *Journal of Nutrition*.

[40] Mukherjee R., Ray C. D., Ray S., Dasgupta S., Chaudhury K. (2014). Altered metabolic profile in early and late onset preeclampsia: An FTIR spectroscopic study. *Pregnancy Hypertension: An International Journal of Women's Cardiovascular Health*.

[41] Kuc S., Koster M. P. H., Pennings J. L. A., Hankemeier T., Berger R. (2014). Metabolomics profiling for identification of novel potential markers in early prediction of preeclampsia. *PLoS ONE*.

[42] Desforges M., Parsons L., Westwood M., Sibley C. P., Greenwood S. L. (2013). Taurine transport in human placental trophoblast is important for regulation of cell differentiation and survival. *Cell Death & Disease*.

[43] Unterscheider J., Daly S., Geary M. P. (2014). Definition and management of fetal growth restriction: A survey of contemporary attitudes. *European Journal of Obstetrics & Gynecology and Reproductive Biology*.

[44] Lausman A., McCarthy F. P., Walker M., Kingdom J. (2013). Screening, diagnosis, and management of intrauterine growth restriction. *Journal of Obstetrics and Gynaecology Canada*.

[45] Maitre L., Fthenou E., Athersuch T. (2014). Urinary metabolic profiles in early pregnancy are associated with preterm birth and fetal growth restriction in the Rhea mother-child cohort study. *BMC Medicine*.

[46] van Vliet E., Eixarch E., Illa M. (2013). Metabolomics Reveals Metabolic Alterations by Intrauterine Growth Restriction in the Fetal Rabbit Brain. *PLoS ONE*.

[47] Horgan R. P., Broadhurst D. I., Walsh S. K. (2011). Metabolic profiling uncovers a phenotypic signature of small for gestational age in early pregnancy. *Journal of Proteome Research*.

[48] Maceyka M., Payne S. G., Milstien S., Spiegel S. (2002). Sphingosine kinase, sphingosine-1-phosphate, and apoptosis. *Biochimica et Biophysica Acta*.

[49] Spiegel S., Milstien S. (2002). Sphingosine 1-phosphate, a key cell signaling molecule. *The Journal of Biological Chemistry*.

[50] Smith S. C., Baker P. N., Symonds E. M. (1997). Increased placental apoptosis in intrauterine growth restriction. *American Journal of Obstetrics & Gynecology*.

[51] Chen C.-P., Bajoria R., Aplin J. D. (2002). Decreased vascularization and cell proliferation in placentas of intrauterine growth-restricted fetuses with abnormal umbilical artery flow velocity waveforms. *American Journal of Obstetrics & Gynecology*.

[52] Tincani A., Cavazzana I., Ziglioli T., Lojacono A., De Angelis V., Meroni P. (2010). Complement activation and pregnancy failure. *Clinical Reviews in Allergy & Immunology*.

[53] Demougeot C., Marie C., Giroud M., Beley A. (2004). N-acetylaspartate: A literature review of animal research on brain ischaemia. *Journal of Neurochemistry*.

[54] Favretto D., Cosmi E., Ragazzi E. (2012). Cord blood metabolomic profiling in intrauterine growth restriction. *Analytical and Bioanalytical Chemistry*.

[55] Goldenberg R. L., Culhane J. F., Iams J. D., Romero R. (2008). Epidemiology and causes of preterm birth. *The Lancet*.

[56] Auray-Blais C., Raiche E., Gagnon R., Berthiaume M., Pasquier J.-C. (2011). Metabolomics and preterm birth: What biomarkers in cervicovaginal secretions are predictive of high-risk pregnant women?. *International Journal of Mass Spectrometry*.

[57] Amabebe E., Reynolds S., Stern V. L. (2016). Identifying metabolite markers for preterm birth in cervicovaginal fluid by magnetic resonance spectroscopy. *Metabolomics*.

[58] Oladipo O. O., Weindel A. L., Saunders A. N., Dietzen D. J. (2011). Impact of premature birth and critical illness on neonatal range of plasma amino acid concentrations determined by LC-MS/MS. *Molecular Genetics and Metabolism*.

[59] Torri G. M., Torri J., Gulian J.-M., Vion-Dury J., Viout P., J. Cozzone P. (1999). Magnetic resonance spectroscopy of serum and acute-phase proteins revisited: A multiparametric statistical analysis of metabolite variations in inflammatory, infectious and miscellaneous diseases. *Clinica Chimica Acta*.

[60] Dessì A., Marincola F. C., Fanos V. (2015). Metabolomics and the great obstetrical syndromes - GDM, PET, and IUGR. *Best Practice & Research Clinical Obstetrics & Gynaecology*.

[61] Huynh J., Xiong G., Bentley-Lewis R. (2014). A systematic review of metabolite profiling in gestational diabetes mellitus. *Diabetologia*.

[62] American Diabetes Association (2015). Classification and diagnosis of diabetes. *Diabetes Care*.

[63] Lehmann R., Friedrich T., Krebiehl G. (2015). Metabolic profiles during an oral glucose tolerance test in pregnant women with and without gestational diabetes. *Experimental and Clinical Endocrinology and Diabetes*.

[64] Liu T., Li J., Xu F. (2016). Comprehensive analysis of serum metabolites in gestational diabetes mellitus by UPLC/Q-TOF-MS. *Analytical and Bioanalytical Chemistry*.

[65] Dudzik D., Zorawski M., Skotnicki M. (2014). Metabolic fingerprint of gestational diabetes mellitus. *Journal of Proteomics*.

[66] Newgard C. B. (2012). Interplay between lipids and branched-chain amino acids in development of insulin resistance. *Cell Metabolism*.

[67] Copps K. D., White M. F. (2012). Regulation of insulin sensitivity by serine/threonine phosphorylation of insulin receptor substrate proteins IRS1 and IRS2. *Diabetologia*.

[68] Würtz P., Mäkinen V.-P., Soininen P. (2012). Metabolic signatures of insulin resistance in 7,098 young adults. *Diabetes*.

[69] Shah S. H., Crosslin D. R., Haynes C. S. (2012). Branched-chain amino acid levels are associated with improvement in insulin resistance with weight loss. *Diabetologia*.

[70] Chen X., Scholl T. O., Leskiw M., Savaille J., Stein T. P. (2010). Differences in maternal circulating fatty acid composition and dietary fat intake in women with gestational diabetes mellitus or mild gestational hyperglycemia. *Diabetes Care*.

[71] Ebbesson S. O. E., Tejero M. E., López-Alvarenga J. C. (2010). Individual saturated fatty acids are associated with different components of insulin resistance and glucose metabolism: The GOCADAN study. *International Journal of Circumpolar Health*.

[72] Schooneman M. G., Vaz F. M., Houten S. M., Soeters M. R. (2013). Acylcarnitines: Reflecting or inflicting insulin resistance?. *Diabetes*.

[73] Agarwal M. M. (2015). Gestational diabetes mellitus: An update on the current international diagnostic criteria. *World Journal of Diabetes*.

[74] de Seymour J. V., Conlon C. A., Sulek K. (2014). Early pregnancy metabolite profiling discovers a potential biomarker for the subsequent development of gestational diabetes mellitus. *Acta Diabetologica*.

[75] Kintz P. (1992). Gas chromatographic analysis of nicotine and cotinine in hair. *Journal of Chromatography B: Biomedical Sciences and Applications*.

[76] Sachs H., Raff I. (1993). Comparison of quantitative results of drugs in human hair by GC MS. *Forensic Science International*.

[77] Ferguson K. K., McElrath T. F., Ko Y.-A., Mukherjee B., Meeker J. D. (2014). Variability in urinary phthalate metabolite levels across pregnancy and sensitive windows of exposure for the risk of preterm birth. *Environment International*.

[78] Ferguson K. K., McElrath T. F., Meeker J. D. (2014). Environmental phthalate exposure and preterm birth. *JAMA Pediatrics*.

[79] Scott-Goodwin A. C., Puerto M., Moreno I. (2016). Toxic effects of prenatal exposure to alcohol, tobacco and other drugs. *Reproductive Toxicology*.

[80] Swan S. H. (2014). Environmental phthalate exposure and the odds of preterm birth: An important contribution to environmental reproductive epidemiology. *JAMA Pediatrics*.

[81] Ko T. J., Tsai L. Y., Chu L. C., Yeh S. J., Leung C. (2014). Parental smoking during pregnancy and its association with low birth weight, small for gestational age, and preterm birth offspring: A birth cohort study. *Pediatrics and Neonatology*.

[82] Shapiro G. D., Dodds L., Arbuckle T. E. (2015). Exposure to phthalates, bisphenol A and metals in pregnancy and the association with impaired glucose tolerance and gestational diabetes mellitus: The MIREC study. *Environment International*.

[83] Khalil A., O'Brien P. (2010). Alcohol and pregnancy. *Obstetrics, Gynaecology and Reproductive Medicine *.

[84] McDermott S., Bao W., Aelion C. M., Cai B., Lawson A. B. (2014). Does the metal content in soil around a pregnant woman’s home increase the risk of low birth weight for her infant?. *Environmental Geochemistry and Health*.

[85] Salquèbre G., Schummer C., Millet M., Briand O., Appenzeller B. M. R. (2012). Multi-class pesticide analysis in human hair by gas chromatography tandem (triple quadrupole) mass spectrometry with solid phase microextraction and liquid injection. *Analytica Chimica Acta*.

[86] Tsatsakis A. M., Barbounis M. G., Kavalakis M., Kokkinakis M., Terzi I., Tzatzarakis M. N. (2010). Determination of dialkyl phosphates in human hair for the biomonitoring of Exposure to organophosphate pesticides. *Journal of Chromatography B*.

[87] Varrica D., Tamburo E., Dongarrà G., Sposito F. (2014). Trace elements in scalp hair of children chronically exposed to volcanic activity (Mt. Etna, Italy). *Science of the Total Environment*.

[88] Molina-Villalba I., Lacasaña M., Rodríguez-Barranco M. (2015). Biomonitoring of arsenic, cadmium, lead, manganese and mercury in urine and hair of children living near mining and industrial areas. *Chemosphere*.

[89] Nielsen M. K. K., Johansen S. S., Linnet K. (2015). Evaluation of poly-drug use in methadone-related fatalities using segmental hair analysis. *Forensic Science International*.

[90] Nielsen M. K. K., Johansen S. S., Linnet K. (2014). Pre-analytical and analytical variation of drug determination in segmented hair using ultra-performance liquid chromatography-tandem mass spectrometry. *Forensic Science International*.

[91] Mergler D., Anderson H. A., Chan L. H. M. (2007). Methylmercury exposure and health effects in humans: A worldwide concern. *AMBIO*.

[92] Manduca P., Naim A., Signoriello S. (2014). Specific association of teratogen and toxicant metals in hair of newborns with congenital birth defects or developmentally premature birth in a cohort of couples with documented parental exposure to military attacks: Observational study at Al Shifa Hospital, Gaza, Palestine. *International Journal of Environmental Research and Public Health*.

[93] Wang Y., Chen A., Dietrich K. N., Radcliffe J., Caldwell K. L., Rogan W. J. (2014). Postnatal exposure to methyl mercury and neuropsychological development in 7-year-old urban inner-city children exposed to lead in the United States. *Child Neuropsychology*.

[94] Marques R. C., Bernardi J. V. E., Dórea J. G., De Fatima R Moreira M., Malm O. (2014). Perinatal multiple exposure to neurotoxic (lead, methylmercury, ethylmercury, and aluminum) substances and neurodevelopment at six and 24 months of age. *Environmental Pollution*.

[95] Grandjean P., Weihe P., White R. F., Debes F., Araki S. (1997). Cognitive deficit in 7-year-old children with prenatal exposure to methylmercury. *Neurotoxicology and Teratology*.

[96] Tian L.-L., Zhao Y.-C., Wang X.-C. (2009). Effects of gestational cadmium exposure on pregnancy outcome and development in the offspring at age 4.5 years. *Biological Trace Element Research*.

[97] Liu B., Wu F., Li X. (2011). Arsenic, antimony and bismuth in human hair from potentially exposed individuals in the vicinity of antimony mines in Southwest China. *Microchemical Journal*.

[98] Wu B., Chen T. (2010). Changes in hair arsenic concentration in a population exposed to heavy pollution: Follow-up investigation in Chenzhou City, Hunan Province, Southern China. *Journal of Environmental Sciences*.

[99] Rodrigues J. L., Batista B. L., Nunes J. A., Passos C. J. S., Barbosa F. (2008). Evaluation of the use of human hair for biomonitoring the deficiency of essential and exposure to toxic elements. *Science of the Total Environment*.

[100] Wadhwa S. K., Kazi T. G., Afridi H. I., Talpur F. N. (2015). Interaction between carcinogenic and anti-carcinogenic trace elements in the scalp hair samples of different types of Pakistani female cancer patients. *Clinica Chimica Acta*.

[101] Pirard C., Koppen G., De Cremer K. (2014). Hair mercury and urinary cadmium levels in Belgian children and their mothers within the framework of the COPHES/DEMOCOPHES projects. *Science of the Total Environment*.

[102] Piñeiro A. M., Moreda-Piñeiro J., Alonso-Rodríguez E., López-Mahía P., Muniategui-Lorenzo S., Prada-Rodríguez D. (2013). Arsenic species determination in human scalp hair by pressurized hot water extraction and high performance liquid chromatography-inductively coupled plasma-mass spectrometry. *Talanta*.

[103] Kumtabtim U., Matusch A., Ulhoa Dani S., Siripinyanond A., Sabine Becker J. (2011). Biomonitoring for arsenic, toxic and essential metals in single hair strands by laser ablation inductively coupled plasma mass spectrometry. *International Journal of Mass Spectrometry*.

[104] Dressler V. L., Pozebon D., Mesko M. F. (2010). Biomonitoring of essential and toxic metals in single hair using on-line solution-based calibration in laser ablation inductively coupled plasma mass spectrometry. *Talanta*.

[105] Eastman R. R., Jursa T. P., Benedetti C., Lucchini R. G., Smith D. R. (2013). Hair as a biomarker of environmental manganese exposure. *Environmental Science & Technology*.

[106] Gottardo R., Fanigliulo A., Sorio D., Liotta E., Bortolotti F., Tagliaro F. (2012). Monitoring compliance to therapy during addiction treatments by means of hair analysis for drugs and drug metabolites using capillary zone electrophoresis coupled to time-of-flight mass spectrometry. *Forensic Science International*.

[107] Agius R., Nadulski T., Kahl H.-G. (2010). Validation of a headspace solid-phase microextraction-GC-MS/MS for the determination of ethyl glucuronide in hair according to forensic guidelines. *Forensic Science International*.

[108] Fernández M. D. M. R., Di Fazio V., Wille S. M. R., Kummer N., Samyn N. (2014). A quantitative, selective and fast ultra-high performance liquid chromatography tandem mass spectrometry method for the simultaneous analysis of 33 basic drugs in hair (amphetamines, cocaine, opiates, opioids and metabolites). *Journal of Chromatography B*.

[109] Imbert L., Dulaurent S., Mercerolle M., Morichon J., Lachâtre G., Gaulier J.-M. (2014). Development and validation of a single LC-MS/MS assay following SPE for simultaneous hair analysis of amphetamines, opiates, cocaine and metabolites. *Forensic Science International*.

[110] Tassoni G., Mirtella D., Zampi M. (2014). Hair analysis in order to evaluate drug abuse in driver's license regranting procedures. *Forensic Science International*.

[111] Singer L. T., Eisengart L. J., Minnes S. (2005). Prenatal cocaine exposure and infant cognition. *Infant Behavior & Development*.

[112] Trezza V., Cuomo V., Vanderschuren L. J. M. J. (2008). Cannabis and the developing brain: insights from behavior. *European Journal of Pharmacology*.

[113] Schweitzer J. B., Riggins T., Liang X. (2015). Prenatal drug exposure to illicit drugs alters working memory-related brain activity and underlying network properties in adolescence. *Neurotoxicology and Teratology*.

[114] Mercolini L., Mandrioli R., Protti M., Conti M., Serpelloni G., Raggi M. A. (2013). Monitoring of chronic Cannabis abuse: An LC-MS/MS method for hair analysis. *Journal of Pharmaceutical and Biomedical Analysis*.

[115] Wurst F. M., Kempter C., Seidl S., Alt A. (1999). Glucuronide - A marker of alcohol consumption and a relapse marker with clinical and forensic implications. *Alcohol and Alcoholism*.

[116] Pragst F., Auwaerter V., Sporkert F., Spiegel K. (2001). Analysis of fatty acid ethyl esters in hair as possible markers of chronically elevated alcohol consumption by headspace solid-phase microextraction (HS-SPME) and gas chromatography-mass spectrometry (GC-MS). *Forensic Science International*.

[117] Wurst F. M., Kelso E., Weinmann W., Pragst F., Yegles M., Sundström Poromaa I. (2008). Measurement of direct ethanol metabolites suggests higher rate of alcohol use among pregnant women than found with the AUDIT-a pilot study in a population-based sample of Swedish women. *American Journal of Obstetrics & Gynecology*.

[118] Agius R., Nadulski T., Kahl H.-G., Dufaux B. (2012). Ethyl glucuronide in hair - A highly effective test for the monitoring of alcohol consumption. *Forensic Science International*.

[119] Pirro V., Di Corcia D., Seganti F., Salomone A., Vincenti M. (2013). Determination of ethyl glucuronide levels in hair for the assessment of alcohol abstinence. *Forensic Science International*.

[120] Cappelle D., Neels H., Yegles M. (2015). Gas chromatographic determination of ethyl glucuronide in hair: Comparison between tandem mass spectrometry and single quadrupole mass spectrometry. *Forensic Science International*.

[121] Kintz P. (2017). Consensus for the use of alcohol markers in hair for assessment of both abstinence and chronic excessive alcohol consumption. *Forensic Science International*.

[122] Yegles M., Labarthe A., Auwärter V. (2004). Comparison of ethyl glucuronide and fatty acid ethyl ester concentrations in hair of alcoholics, social drinkers and teetotallers. *Forensic Science International*.

[123] Yegles M. (2002). 10ème Congrès Annuel de la SFTA et 6èmes Journées Scientifiques du Centre de Compétence en Chimie et Toxicologie Analytiques - Martigny (Suisse), 19-21 Juin 2002. *Annales de Toxicologie Analytique*.

[124] Auwärter V., Sporkert F., Hartwig S., Pragst F., Vater H., Diefenbacher A. (2001). Fatty acid ethyl esters in hair as markers of alcohol consumption. Segmental hair analysis of alcoholics, social drinkers, and teetotalers. *Clinical Chemistry*.

[125] Tsanaclis L., Kingston R., Wicks J. (2009). Testing for alcohol use in hair: is ethyl glucuronide (EtG) stable in hair?. *Annales de Toxicologie Analytique*.

[126] Crunelle C. L., Yegles M., De Doncker M. (2015). Influence of repeated permanent coloring and bleaching on ethyl glucuronide concentrations in hair from alcohol-dependent patients. *Forensic Science International*.

[127] Suesse S., Pragst F., Mieczkowski T. (2012). Practical experiences in application of hair fatty acid ethyl esters and ethyl glucuronide for detection of chronic alcohol abuse in forensic cases. *Forensic Science International*.

[128] Hartwig S., Auwärter V., Pragst F. (2003). Effect of hair care and hair cosmetics on the concentrations of fatty acid ethyl esters in hair as markers of chronically elevated alcohol consumption. *Forensic Science International*.

[129] Pragst F., Rothe M., Moench B., Hastedt M., Herre S., Simmert D. (2010). Combined use of fatty acid ethyl esters and ethyl glucuronide in hair for diagnosis of alcohol abuse: Interpretation and advantages. *Forensic Science International*.

[130] Appenzeller B. M. R., Mathon C., Schummer C., Alkerwi A., Lair M.-L. (2012). Simultaneous determination of nicotine and PAH metabolites in human hair specimen: A potential methodology to assess tobacco smoke contribution in PAH exposure. *Toxicology Letters*.

[131] Dow J., Hall K. (1978). Capillary-column combined gas chromatography-mass spectrometry method for the estimation of nicotine in plasma by selective ion monitoring. *Journal of Chromatography A*.

[132] Horning E. C., Horning M. G., Carroll D. I., Stillwell R. N., Dzidic I. (1973). Nicotine in smokers, non-smokers and room air. *Life Sciences*.

[133] Mahoney G. N., Al-Delaimy W. (2001). Measurement of nicotine in hair by reversed-phase high-performance liquid chromatography with electrochemical detection. *Journal of Chromatography B: Biomedical Sciences and Applications*.

[134] Yang J., Hu Y., Cai J. B. (2007). Selective hair analysis of nicotine by molecular imprinted solid-phase extraction: An application for evaluating tobacco smoke exposure. *Food and Chemical Toxicology*.

[135] Man C. N., Ismail S., Harn G. L., Lajis R., Awang R. (2009). Determination of hair nicotine by gas chromatography-mass spectrometry. *Journal of Chromatography B*.

[136] Europe P. (2008). *Which Pesticides are Banned in Europe*.

[137] WHO (2010). *Expsoure to Highly Hazardous Pesticides: A Major Public Health Concern*.

[138] Levario-Carrillo M., Amato D., Ostrosky-Wegman P., González-Horta C., Corona Y., Sanin L. H. (2004). Relation between pesticide exposure and intrauterine growth retardation. *Chemosphere*.

[139] Weselak M., Arbuckle T. E., Wigle D. T., Walker M. C., Krewski D. (2008). Pre- and post-conception pesticide exposure and the risk of birth defects in an Ontario farm population. *Reproductive Toxicology*.

[140] Chand S., Mustafa M. D., Banerjee B. D., Guleria K. (2015). CYP17A1 gene polymorphisms and environmental exposure to organochlorine pesticides contribute to the risk of small for gestational age. *European Journal of Obstetrics & Gynecology and Reproductive Biology*.

[141] González-Alzaga B., Hernández A. F., Rodríguez-Barranco M. (2015). Pre- and postnatal exposures to pesticides and neurodevelopmental effects in children living in agricultural communities from South-Eastern Spain. *Environment International*.

[142] Michalakis M., Tzatzarakis M. N., Kovatsi L. (2014). Hypospadias in offspring is associated with chronic exposure of parents to organophosphate and organochlorine pesticides. *Toxicology Letters*.

[143] Lu D., Feng C., Lin Y. (2014). Determination of organochlorines, polychlorinated biphenyls and polybrominated diphenyl ethers in human hair: Estimation of external and internal exposure. *Chemosphere*.

[144] Duca R.-C., Salquebre G., Hardy E., Appenzeller B. M. R. (2014). Comparison of solid phase- and liquid/liquid-extraction for the purification of hair extract prior to multi-class pesticides analysis. *Journal of Chromatography B*.

[145] Sulek K., Han T.-L., Villas-Boas S. G. R. (2014). Hair metabolomics: identification of fetal compromise provides proof of concept for biomarker discovery. *Theranostics*.

[146] He X., de Seymour J. V., Sulek K. (2016). Maternal hair metabolome analysis identifies a potential marker of lipid peroxidation in gestational diabetes mellitus. *Acta Diabetologica*.

[147] Yoshioka K., Shimojo N., Nakanishi T., Naka K., Okuda K. (1994). Measurements of urinary adipic acid and suberic acid using high-performance liquid chromatography. *Journal of Chromatography B: Biomedical Sciences and Applications*.

[148] Pettersen J. E., Jeelum E., Eedjarn L. (1972). The occurrence of adipic and suberic acid in urine from ketotic patients. *Clinica Chimica Acta*.

[149] Inouye M., Mio T., Sumino K. (2000). Dicarboxylic acids as markers of fatty acid peroxidation in diabetes. *Atherosclerosis*.

[150] Wild C. P. (2005). Complementing the genome with an "exposome": The outstanding challenge of environmental exposure measurement in molecular epidemiology. *Cancer Epidemiology, Biomarkers & Prevention*.

[151] Nakamura J., Mutlu E., Sharma V. (2014). The endogenous exposome. *DNA Repair*.

[152] Shapiro G. D., Dodds L., Arbuckle T. E. (2016). Exposure to organophosphorus and organochlorine pesticides, perfluoroalkyl substances, and polychlorinated biphenyls in pregnancy and the association with impaired glucose tolerance and gestational diabetes mellitus: The MIREC Study. *Environmental Research*.

[153] Sakamoto M., Chan H. M., Domingo J. L., Kawakami S., Murata K. (2012). Mercury and docosahexaenoic acid levels in maternal and cord blood in relation to segmental maternal hair mercury concentrations at parturition. *Environment International*.

[154] Ostrea Jr E. M., Bielawski D. M., Posecion N. C. (2008). A comparison of infant hair, cord blood and meconium analysis to detect fetal exposure to environmental pesticides. *Environmental Research*.

[155] Ostrea Jr E. M., Villanueva-Uy E., Bielawski D. M. (2006). Maternal hair-An appropriate matrix for detecting maternal exposure to pesticides during pregnancy. *Environmental Research*.

[156] Posecion Jr N., Ostrea Jr E., Bielawski D., Corrion M., Seagraves J., Jin Y. (2006). Detection of exposure to environmental pesticides during pregnancy by the analysis of maternal hair using GC-MS. *Chromatographia*.

[157] Basu N., Tutino R., Zhang Z. (2014). Mercury levels in pregnant women, children, and seafood from Mexico City. *Environmental Research*.

[158] Nunes E., Cavaco A., Carvalho C. (2014). Exposure assessment of pregnant portuguese women to methylmercury through the ingestion of fish: Cross-sectional survey and biomarker validation. *Journal of Toxicology and Environmental Health, Part A. Current Issues*.

[159] Ostrea Jr E. M., Reyes A., Villanueva-Uy E. (2012). Fetal exposure to propoxur and abnormal child neurodevelopment at 2 years of age. *NeuroToxicology*.

[160] Corrion M. L., Ostrea E. M., Bielawski D. M., Posecion Jr N. C., Seagraves J. J. (2005). Detection of prenatal exposure to several classes of environmental toxicants and their metabolites by gas chromatography-mass spectrometry in maternal and umbilical cord blood. *Journal of Chromatography B*.

[161] Klein J., Blanchette P., Koren G. (2004). Assessing nicotine metabolism in pregnancy - A novel approach using hair analysis. *Forensic Science International*.

[162] Falcon M., Pichini S., Joya J. (2012). Maternal hair testing for the assessment of fetal exposure to drug of abuse during early pregnancy: Comparison with testing in placental and fetal remains. *Forensic Science International*.

[163] Ostrea Jr E. M., Hernandez J. D., Bielawski D. M. (2006). Fatty acid ethyl esters in meconium: Are they biomarkers of fetal alcohol exposure and effect?. *Alcoholism: Clinical and Experimental Research*.

[164] Bonvallot N., Tremblay-Franco M., Chevrier C. (2013). Metabolomics tools for describing complex pesticide exposure in pregnant women in brittany (France). *PLoS ONE*.

[165] Moreno Villares J. M. (2016). Nutrition in early life and the programming of adult disease: the first 1000 days. *Nutrición Hospitalaria*.

[166] Mothers SotWs Nutrition in the First 1,000 Days. Save the Children.

[167] Kenny L. C., Dunn W. B., Ellis D. I., Myers J., Baker P. N., Kell D. B. (2005). Novel biomarkers for pre-eclampsia detected using metabolomics and machine learning. *Metabolomics*.

[168] Kenny L. C., Broadhurst D., Brown M. (2008). Detection and identification of novel metabolomic biomarkers in preeclampsia. *Reproductive Sciences*.

[169] Kenny L. C., Broadhurst D. I., Dunn W. (2010). Robust early pregnancy prediction of later preeclampsia using metabolomic biomarkers. *Hypertension*.

[170] Dunn W. B., Brown M., Worton S. A. (2012). The metabolome of human placental tissue: Investigation of first trimester tissue and changes related to preeclampsia in late pregnancy. *Metabolomics*.

[171] Bahado-Singh R. O., Akolekar R., Mandal R. (2012). Metabolomics and first-trimester prediction of early-onset preeclampsia. *The Journal of Maternal-Fetal and Neonatal Medicine*.

[172] Bahado-Singh R. O., Akolekar R., Mandal R. (2013). First-trimester metabolomic detection of late-onset preeclampsia. *American Journal of Obstetrics & Gynecology*.

[173] Bahado-Singh R. O., Syngelaki A., Akolekar R. (2015). Validation of metabolomic models for prediction of early-onset preeclampsia. *American Journal of Obstetrics & Gynecology*.

[174] Odibo A. O., Goetzinger K. R., Odibo L. (2011). First-trimester prediction of preeclampsia using metabolomic biomarkers: a discovery phase study. *Prenatal Diagnosis*.

[175] Austdal M., Skrastad R. B., Gundersen A. S., Austgulen R., Iversen A.-C., Bathen T. F. (2014). Metabolomic biomarkers in serum and urine in women with preeclampsia. *PLoS ONE*.

[176] Austdal M., Tangeras L. H., Skrastad R. B. (2015). First trimester urine and serum metabolomics for prediction of preeclampsia and gestational hypertension: A prospective screening study. *International Journal of Molecular Sciences*.

[177] Dessì A., Atzori L., Noto A., Adriaan Visser G. H., Gazzolo D. (2011). Metabolomics in newborns with intrauterine growth retardation (IUGR): urine reveals markers of metabolic syndrome. *The Journal of Maternal-Fetal & Neonatal Medicine*.

[178] Hajduk J., Klupczynska A., Dereziński P. (2015). A combined metabolomic and proteomic analysis of gestational diabetes mellitus. *International Journal of Molecular Sciences*.

[179] Enquobahrie D. A., Denis M., Tadesse M. G., Gelaye B., Ressom H. W., Williams M. A. (2015). Maternal early pregnancy serum metabolites and risk of gestational diabetes mellitus. *The Journal of Clinical Endocrinology & Metabolism*.

[180] Bentley-Lewis R., Huynh J., Xiong G. (2015). Metabolomic profiling in the prediction of gestational diabetes mellitus. *Diabetologia*.

[181] Sachse D., Sletner L., Mørkrid K. (2012). Metabolic Changes in Urine during and after Pregnancy in a Large, Multiethnic Population-Based Cohort Study of Gestational Diabetes. *PLoS ONE*.

